# Bis(3-methyl­anilinium) naphthalene-1,5-disulfonate

**DOI:** 10.1107/S1600536812021290

**Published:** 2012-05-16

**Authors:** Ming-Liang Liu, Zi-Qi Chen

**Affiliations:** a Ordered Matter Science Research Center, Southeast University, Nanjing 211189, People’s Republic of China

## Abstract

In the crystal of the title mol­ecular salt, 2C_7_H_10_N^+^·C_10_H_6_O_6_S_2_^2−^, the naphthalene-1,5-disulfonate anion is located on an inversion center and accepts N—H⋯O hydrogen bonds from the 3-methyl­anilinium cations, forming supra­molecular layers parallel to the *ac* plane.

## Related literature

For background to ferroelectric compounds, see: Fu *et al.* (2011 ▶); Ye *et al.* (2009 ▶); Zhang & Xiong (2012 ▶); Zhang *et al.* (2009 ▶, 2010 ▶). For a related structure, see: Liu (2012 ▶). 
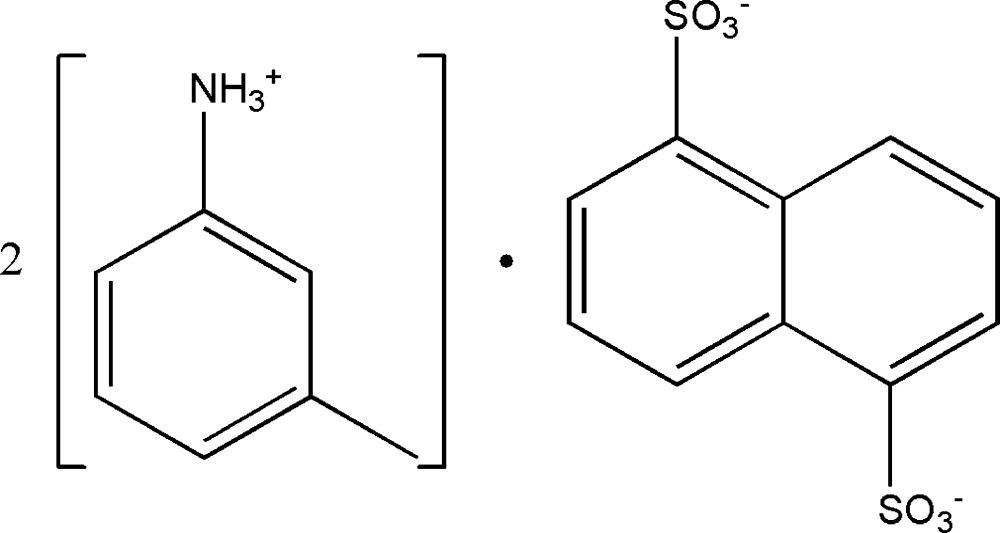


## Experimental

### 

#### Crystal data

2C_7_H_10_N^+^·C_10_H_6_O_6_S_2_^2−^
*M* *_r_* = 502.59
Monoclinic, 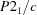

*a* = 8.3426 (17) Å
*b* = 19.896 (4) Å
*c* = 7.0670 (14) Å
β = 90.14 (3)°
*V* = 1173.0 (4) Å^3^
*Z* = 2
Mo *K*α radiation
μ = 0.27 mm^−1^
*T* = 293 K
0.36 × 0.32 × 0.28 mm

#### Data collection

Rigaku Mercury2 diffractometer
Absorption correction: multi-scan (*CrystalClear*; Rigaku, 2005 ▶) *T*_min_ = 0.901, *T*_max_ = 0.923
10755 measured reflections
2311 independent reflections
2131 reflections with *I* > 2σ(*I*)
*R*_int_ = 0.036

#### Refinement

*R*[*F* ^2^ > 2σ(*F* ^2^)] = 0.082
*wR*(*F* ^2^) = 0.190
*S* = 1.22
2311 reflections
156 parameters
H-atom parameters constrained
Δρ_max_ = 0.50 e Å^−3^
Δρ_min_ = −0.33 e Å^−3^

### 

Data collection: *CrystalClear* (Rigaku, 2005 ▶); cell refinement: *CrystalClear*; data reduction: *CrystalClear*; program(s) used to solve structure: *SHELXTL* (Sheldrick, 2008 ▶); program(s) used to refine structure: *SHELXTL*; molecular graphics: *SHELXTL*; software used to prepare material for publication: *SHELXTL*.

## Supplementary Material

Crystal structure: contains datablock(s) I, global. DOI: 10.1107/S1600536812021290/xu5533sup1.cif

Structure factors: contains datablock(s) I. DOI: 10.1107/S1600536812021290/xu5533Isup2.hkl

Supplementary material file. DOI: 10.1107/S1600536812021290/xu5533Isup3.cml

Additional supplementary materials:  crystallographic information; 3D view; checkCIF report

## Figures and Tables

**Table 1:** Hydrogen-bond geometry (Å, °)

*D*—H⋯*A*	*D*—H	H⋯*A*	*D*⋯*A*	*D*—H⋯*A*
N1—H1*A*⋯O3^i^	0.89	1.90	2.779 (6)	168
N1—H1*B*⋯O2^ii^	0.89	1.89	2.779 (6)	177
N1—H1*C*⋯O1^iii^	0.89	1.93	2.797 (6)	165

Symmetry codes: (i) 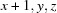; (ii) 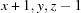; (iii) 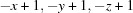.

**Fig. 1.**
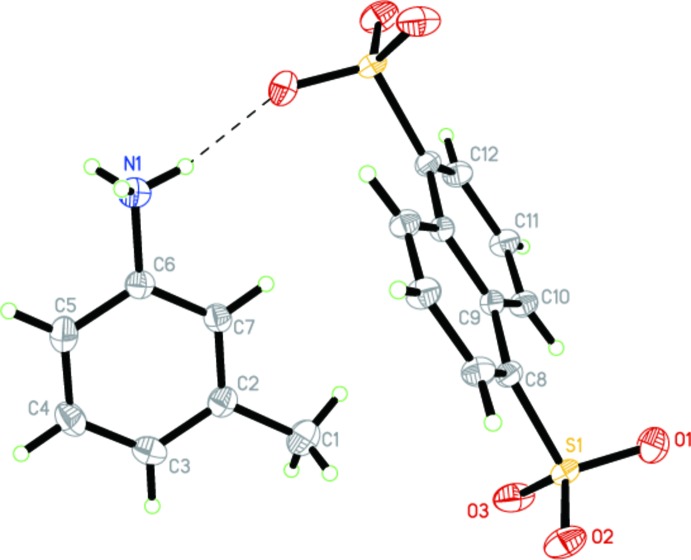
The molecular structure of the title compound, showing the atomic numbering scheme with 30% probability displacement ellipsoids. The unlablled atoms are in the asymmetric unit at (1-x,1-y,1-z).

**Fig. 2.**
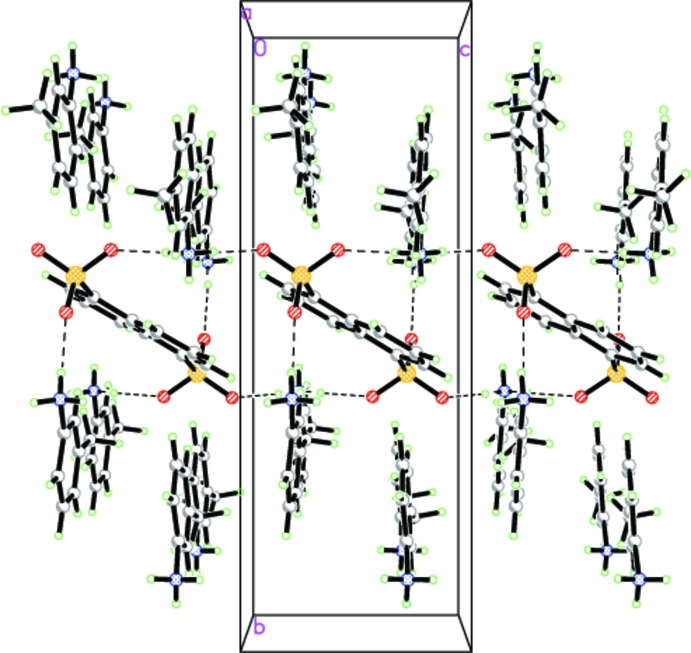
The packing of the title compound with view along the a axis. Dashed lines indicate hydrogen bonds.

## supplementary crystallographic information

### Comment

Recently much attention has been devoted to simple molecular-ionic compounds containing inorganic and organic ions due to the tunability of their special structural features and their potential ferroelectrics property. Ferroelectric materials that exhibit reversible electric polarization in response to an external electric field have found many applications such as nonvolatile memory storage, electronics and optics. The freezing of a certain functional group at low temperature forces significant orientational motions of the guest molecules and thus induces the formation of the ferroelectric phase. (Fu *et al*, 2011; Ye *et al.* 2009; Zhang *et al.* 2009; Zhang & Xiong, 2012; Zhang *et al.* 2010). In our laboratory, the title compound has been synthesized to investigate to its potential ferroelectric properties. However, it was found that the dielectric constant of the compound as a function of temperature indicates that the permittivity is basically temperature-independent (*ε* = C/(T–T_0_)), suggesting that this compound is not ferroelectric or there may be no distinct phase transition occurring within the measured temperature (below the melting point).

The title compound,(C_7_H_10_N)_2_.C_10_H_6_O_6_S_2_, has an asymmetric unit that consists of 3-methylanilinium cation, half an naphthalene-1,5-disulfonate anions, which are linked by an N—H···O hydrogen bond(Fig 1). The non-hydrogen atoms of the cation and the anion are coplanar with the r.m.s deviation are 0.0123Å and 0.0326Å respectively, the angle of the two plane is 114.7°. In the crystal structure, the cations are linked to anions by N—H···O hydrogen bonds to form layer-like structure which is parallel to *ac* plane (Fig 2).

### Experimental

1.07 g (1 mmol) of 3-toluidine was firstly dissolved in 30 ml of ethanol, to which 0.288 g (1 mmol) of 1,5-naphthalene-disulfonic acid was added to give a solution at the ambient temperature. Single crystals suitable for X-ray structure analysis were obtained by the slow evaporation of the above solution after 5 days in air.

### Refinement

H atoms were placed in calculated positions with N—H = 0.89 and C—H = 0.93– 0.96 Å, and refined in riding mode, U_iso_(H) = 1.5U_eq_(C,N) for methyl and amino H atoms and 1.2U_eq_(C) for the others.

### Crystal data

**Table:**

2C_7_H_10_N^+^·C_10_H_6_O_6_S_2_^2^^−^	*F*(000) = 528
*M**_r_* = 502.59	*D*_x_ = 1.423 Mg m^−^^3^
Monoclinic, *P*2_1_/*c*	Mo *K*α radiation, λ = 0.71073 Å
Hall symbol: -P 2ybc	Cell parameters from 2066 reflections
*a* = 8.3426 (17) Å	θ = 3.4–25.0°
*b* = 19.896 (4) Å	µ = 0.27 mm^−^^1^
*c* = 7.0670 (14) Å	*T* = 293 K
β = 90.14 (3)°	Block, colourless
*V* = 1173.0 (4) Å^3^	0.36 × 0.32 × 0.28 mm
*Z* = 2	

### Data collection

**Table:**

Rigaku Mercury2 diffractometer	2311 independent reflections
Radiation source: fine-focus sealed tube	2131 reflections with *I* > 2σ(*I*)
Graphite monochromator	*R*_int_ = 0.036
Detector resolution: 13.6612 pixels mm^-1^	θ_max_ = 26.0°, θ_min_ = 3.1°
ω scans	*h* = −10→10
Absorption correction: multi-scan (*CrystalClear*; Rigaku, 2005)	*k* = −24→24
*T*_min_ = 0.901, *T*_max_ = 0.923	*l* = −8→8
10755 measured reflections	

### Refinement

**Table:**

Refinement on *F*^2^	Primary atom site location: structure-invariant direct methods
Least-squares matrix: full	Secondary atom site location: difference Fourier map
*R*[*F*^2^ > 2σ(*F*^2^)] = 0.082	Hydrogen site location: inferred from neighbouring sites
*wR*(*F*^2^) = 0.190	H-atom parameters constrained
*S* = 1.22	*w* = 1/[σ^2^(*F*_o_^2^) + (0.0077*P*)^2^ + 6.1071*P*] where *P* = (*F*_o_^2^ + 2*F*_c_^2^)/3
2311 reflections	(Δ/σ)_max_ = 0.034
156 parameters	Δρ_max_ = 0.50 e Å^−^^3^
0 restraints	Δρ_min_ = −0.33 e Å^−^^3^

### Special details

**Table:**

Geometry. All esds (except the esd in the dihedral angle between two l.s. planes) are estimated using the full covariance matrix. The cell esds are taken into account individually in the estimation of esds in distances, angles and torsion angles; correlations between esds in cell parameters are only used when they are defined by crystal symmetry. An approximate (isotropic) treatment of cell esds is used for estimating esds involving l.s. planes.
Refinement. Refinement of F^2^ against ALL reflections. The weighted R-factor wR and goodness of fit S are based on F^2^, conventional R-factors R are based on F, with F set to zero for negative F^2^. The threshold expression of F^2^ > 2sigma(F^2^) is used only for calculating R-factors(gt) etc. and is not relevant to the choice of reflections for refinement. R-factors based on F^2^ are statistically about twice as large as those based on F, and R- factors based on ALL data will be even larger.

### Fractional atomic coordinates and isotropic or equivalent isotropic displacement parameters (Å^2^)

**Table:**

	*x*	*y*	*z*	*U*_iso_*/*U*_eq_	
S1	0.19559 (14)	0.57953 (6)	0.74214 (17)	0.0295 (3)	
O2	0.2105 (5)	0.6206 (2)	0.9102 (5)	0.0493 (11)	
O3	0.1516 (5)	0.6197 (2)	0.5795 (6)	0.0518 (11)	
O1	0.0920 (5)	0.5221 (2)	0.7674 (7)	0.0554 (12)	
N1	1.0108 (5)	0.6117 (2)	0.2234 (6)	0.0388 (10)	
H1A	1.0688	0.6144	0.3290	0.058*	
H1B	1.0748	0.6160	0.1236	0.058*	
H1C	0.9618	0.5720	0.2189	0.058*	
C11	0.3285 (6)	0.4582 (3)	0.2293 (7)	0.0360 (12)	
H11	0.2464	0.4479	0.1447	0.043*	
C8	0.3919 (5)	0.5474 (2)	0.6913 (7)	0.0288 (10)	
C9	0.4199 (5)	0.5100 (2)	0.5201 (6)	0.0265 (10)	
C7	0.7308 (6)	0.6503 (3)	0.2401 (7)	0.0334 (11)	
H7	0.6988	0.6057	0.2505	0.040*	
C10	0.2950 (6)	0.4918 (2)	0.3920 (7)	0.0317 (11)	
H10	0.1895	0.5031	0.4200	0.038*	
C6	0.8907 (6)	0.6655 (3)	0.2215 (7)	0.0331 (11)	
C2	0.6166 (6)	0.7012 (3)	0.2436 (7)	0.0374 (12)	
C12	0.4886 (6)	0.4387 (3)	0.1880 (7)	0.0360 (11)	
H12	0.5106	0.4160	0.0759	0.043*	
C5	0.9429 (7)	0.7315 (3)	0.2022 (8)	0.0441 (13)	
H5	1.0511	0.7413	0.1871	0.053*	
C3	0.6696 (7)	0.7671 (3)	0.2287 (8)	0.0434 (13)	
H3	0.5954	0.8019	0.2342	0.052*	
C4	0.8297 (8)	0.7822 (3)	0.2060 (8)	0.0461 (14)	
H4	0.8617	0.8267	0.1933	0.055*	
C1	0.4405 (7)	0.6864 (4)	0.2685 (11)	0.0618 (18)	
H1D	0.4111	0.6935	0.3982	0.093*	
H1E	0.4196	0.6404	0.2346	0.093*	
H1F	0.3787	0.7156	0.1886	0.093*	

### Atomic displacement parameters (Å^2^)

**Table:**

	*U*^11^	*U*^22^	*U*^33^	*U*^12^	*U*^13^	*U*^23^
S1	0.0236 (5)	0.0366 (6)	0.0282 (6)	0.0052 (5)	0.0023 (4)	−0.0040 (5)
O2	0.050 (2)	0.064 (3)	0.034 (2)	0.007 (2)	0.0046 (17)	−0.0176 (19)
O3	0.063 (3)	0.055 (3)	0.037 (2)	0.026 (2)	0.0026 (19)	0.0006 (19)
O1	0.033 (2)	0.051 (3)	0.082 (3)	−0.0064 (18)	0.012 (2)	0.000 (2)
N1	0.042 (2)	0.038 (2)	0.037 (2)	0.0038 (19)	0.0001 (19)	−0.0004 (19)
C11	0.026 (2)	0.042 (3)	0.039 (3)	−0.001 (2)	−0.006 (2)	−0.012 (2)
C8	0.026 (2)	0.031 (2)	0.030 (2)	0.0020 (19)	0.0028 (19)	−0.0006 (19)
C9	0.027 (2)	0.026 (2)	0.027 (2)	0.0007 (18)	0.0021 (18)	0.0014 (18)
C7	0.040 (3)	0.032 (3)	0.028 (2)	−0.004 (2)	0.002 (2)	−0.004 (2)
C10	0.026 (2)	0.037 (3)	0.033 (3)	0.003 (2)	−0.0034 (19)	−0.002 (2)
C6	0.037 (3)	0.034 (3)	0.027 (2)	0.003 (2)	0.002 (2)	0.002 (2)
C2	0.038 (3)	0.045 (3)	0.029 (3)	−0.002 (2)	0.001 (2)	−0.003 (2)
C12	0.038 (3)	0.037 (3)	0.033 (3)	0.004 (2)	0.001 (2)	−0.006 (2)
C5	0.042 (3)	0.048 (3)	0.042 (3)	−0.011 (3)	0.004 (2)	0.001 (3)
C3	0.051 (3)	0.035 (3)	0.044 (3)	0.007 (2)	−0.003 (3)	−0.001 (2)
C4	0.061 (4)	0.033 (3)	0.045 (3)	−0.006 (3)	0.001 (3)	0.006 (2)
C1	0.037 (3)	0.066 (4)	0.083 (5)	−0.002 (3)	0.007 (3)	−0.002 (4)

### Geometric parameters (Å, º)

**Table:**

S1—O1	1.444 (4)	C7—C2	1.392 (7)
S1—O2	1.446 (4)	C7—H7	0.9300
S1—O3	1.446 (4)	C10—H10	0.9300
S1—C8	1.795 (5)	C6—C5	1.391 (7)
N1—C6	1.467 (6)	C2—C3	1.387 (8)
N1—H1A	0.8900	C2—C1	1.509 (8)
N1—H1B	0.8900	C12—C8^i^	1.339 (7)
N1—H1C	0.8900	C12—H12	0.9300
C11—C10	1.360 (7)	C5—C4	1.381 (8)
C11—C12	1.422 (7)	C5—H5	0.9300
C11—H11	0.9300	C3—C4	1.379 (8)
C8—C12^i^	1.339 (7)	C3—H3	0.9300
C8—C9	1.440 (6)	C4—H4	0.9300
C9—C10	1.425 (6)	C1—H1D	0.9600
C9—C9^i^	1.424 (9)	C1—H1E	0.9600
C7—C6	1.374 (7)	C1—H1F	0.9600
			
O1—S1—O2	113.3 (3)	C9—C10—H10	119.7
O1—S1—O3	112.6 (3)	C7—C6—C5	121.4 (5)
O2—S1—O3	111.3 (2)	C7—C6—N1	120.2 (5)
O1—S1—C8	106.8 (2)	C5—C6—N1	118.5 (5)
O2—S1—C8	106.8 (2)	C3—C2—C7	117.9 (5)
O3—S1—C8	105.5 (2)	C3—C2—C1	120.3 (5)
C6—N1—H1A	109.5	C7—C2—C1	121.7 (5)
C6—N1—H1B	109.5	C8^i^—C12—C11	120.7 (5)
H1A—N1—H1B	109.5	C8^i^—C12—H12	119.6
C6—N1—H1C	109.5	C11—C12—H12	119.6
H1A—N1—H1C	109.5	C4—C5—C6	118.3 (5)
H1B—N1—H1C	109.5	C4—C5—H5	120.9
C10—C11—C12	120.2 (5)	C6—C5—H5	120.9
C10—C11—H11	119.9	C4—C3—C2	121.6 (5)
C12—C11—H11	119.9	C4—C3—H3	119.2
C12^i^—C8—C9	121.3 (4)	C2—C3—H3	119.2
C12^i^—C8—S1	118.5 (4)	C3—C4—C5	120.4 (5)
C9—C8—S1	120.1 (3)	C3—C4—H4	119.8
C10—C9—C9^i^	119.2 (5)	C5—C4—H4	119.8
C10—C9—C8	123.0 (4)	C2—C1—H1D	109.5
C9^i^—C9—C8	117.8 (5)	C2—C1—H1E	109.5
C6—C7—C2	120.5 (5)	H1D—C1—H1E	109.5
C6—C7—H7	119.8	C2—C1—H1F	109.5
C2—C7—H7	119.8	H1D—C1—H1F	109.5
C11—C10—C9	120.7 (4)	H1E—C1—H1F	109.5
C11—C10—H10	119.7		

Symmetry code: (i) −*x*+1, −*y*+1, −*z*+1.

### Hydrogen-bond geometry (Å, º)

**Table:**

*D*—H···*A*	*D*—H	H···*A*	*D*···*A*	*D*—H···*A*
N1—H1*A*···O3^ii^	0.89	1.90	2.779 (6)	168
N1—H1*B*···O2^iii^	0.89	1.89	2.779 (6)	177
N1—H1*C*···O1^i^	0.89	1.93	2.797 (6)	165

Symmetry codes: (i) −*x*+1, −*y*+1, −*z*+1; (ii) *x*+1, *y*, *z*; (iii) *x*+1, *y*, *z*−1.
